# NCAM1 Polysialylation: The Prion Protein's Elusive Reason for Being?

**DOI:** 10.1177/1759091416679074

**Published:** 2016-11-22

**Authors:** Mohadeseh Mehrabian, Herbert Hildebrandt, Gerold Schmitt-Ulms

**Affiliations:** 1 Tanz Centre for Research in Neurodegenerative Diseases, University of Toronto, Toronto, Ontario, Canada; 2 Department of Laboratory Medicine and Pathobiology, University of Toronto, Toronto, Ontario, Canada; 3 Institute for Cellular Chemistry, Hannover Medical School, Hannover, Germany

**Keywords:** neural cell adhesion molecules, polysialic acid, prion protein, polysialyltransferases, signaling, protein function

## Abstract

Much confusion surrounds the physiological function of the cellular prion protein (PrP^C^). It is, however, anticipated that knowledge of its function will shed light on its contribution to neurodegenerative diseases and suggest ways to interfere with the cellular toxicity central to them. Consequently, efforts to elucidate its function have been all but exhaustive. Building on earlier work that uncovered the evolutionary descent of the prion founder gene from an ancestral ZIP zinc transporter, we recently investigated a possible role of PrP^C^ in a morphogenetic program referred to as epithelial-to-mesenchymal transition (EMT). By capitalizing on PrP^C^ knockout cell clones in a mammalian cell model of EMT and using a comparative proteomics discovery strategy, neural cell adhesion molecule-1 emerged as a protein whose upregulation during EMT was perturbed in PrP^C^ knockout cells. Follow-up work led us to observe that PrP^C^ regulates the polysialylation of the neural cell adhesion molecule NCAM1 in cells undergoing morphogenetic reprogramming. In addition to governing cellular migration, polysialylation modulates several other cellular plasticity programs PrP^C^ has been phenotypically linked to. These include neurogenesis in the subventricular zone, controlled mossy fiber sprouting and trimming in the hippocampal formation, hematopoietic stem cell renewal, myelin repair and maintenance, integrity of the circadian rhythm, and glutamatergic signaling. This review revisits this body of literature and attempts to present it in light of this novel contextual framework. When approached in this manner, a coherent model of PrP^C^ acting as a regulator of polysialylation during specific cell and tissue morphogenesis events comes into focus.

## Background

Arguably the most successful strategies for elucidating the function of a protein are (a) to extrapolate it from the known function of its binding partners, (b) to interpret the phenotypic consequences of deleting the gene coding for it, or (c) to derive it by studying the function of homologous proteins and place its role in the context of evolution. For the cellular prion protein (PrP^C^), all three approaches have been pursued with moderate successes summarized in comprehensive earlier reviews (Steele et al., 2007; Watts and Westaway, 2007; Aguzzi et al., 2008; Ehsani, Huo, et al., 2011). Once the predominant function of a protein is known, data gathered by all three approaches tend to merge in ways that make sense. Furthermore, as insights into the mechanism of action underlying the function of a protein are refined, useful predictions can often be made about how it will behave when placed into a hitherto unexplored experimental paradigm.

**Figure 1.**
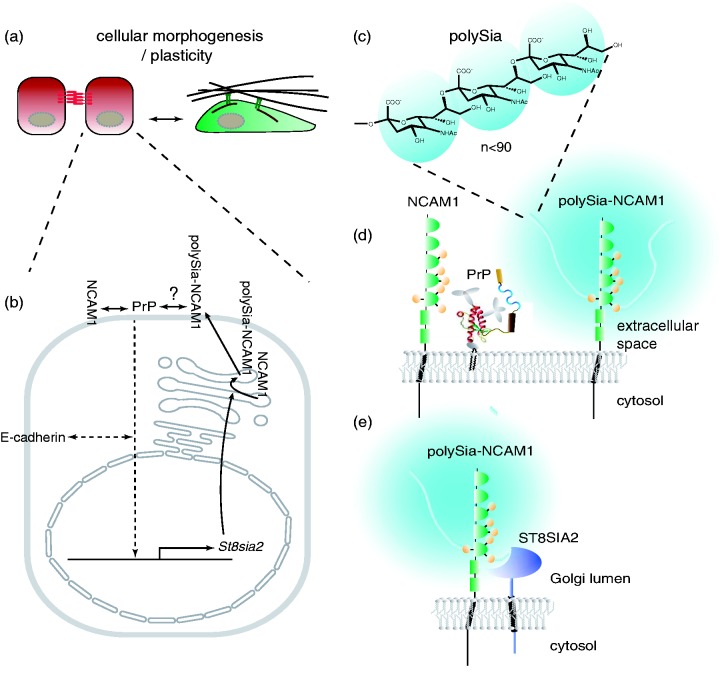
Cartoon depicting PrP-dependent NCAM1 polysialylation. (a) The proposed role for PrP has so far only been observed in the context of specific cellular morphogenesis or plasticity programs. (b) Depiction of molecular components of PrP-ST8SIA2-NCAM1 signaling loop. (c) Chemical structure of polySia homopolymer composed of α2,8-glycosidically linked N-acetylneuraminic acids. (d) Membrane topology and approximate relative sizes of key players. (e) Membrane topology and approximate size of ST8SIA2 relative to NCAM1 in Golgi compartment, the proposed subcellular site in which NCAM1 polysialylation occurs.

The elusive nature of the function of PrP^C^ combined with the large number of reports dedicated to this topic has led to a widespread sentiment that PrP^C^ harbors several independent activities and its function may be context-dependent, multifaceted, or impossible to know with certainty. Although this perspective has merit—and perhaps represents a truism that applies to any protein—adapting this position is not productive for efforts to identify the key role of a protein, which most often eventually comes to the fore. Here, we will take a different view: We will point toward data which suggest that PrP^C^ may operate in several cellular plasticity events as a highly specialized partner of neural cell adhesion molecule-1 (NCAM1). According to this model, PrP^C^ acts upstream of a signaling loop, which modulates the expression of the polysialyltransferase (polyST) ST8SIA2 (and perhaps its paralog ST8SIA4), thereby determining levels of polysialylation of NCAM1 (and, possibly, other polyST substrates), during the execution of specific cell plasticity programs (Figure 1).

A full account of the function of a protein requires an understanding of both the broader cellular context it contributes to and the precise mechanism by which it contributes. It therefore should be stressed that even if the model we are proposing for PrP^C^ will hold up upon close scrutiny, more work is needed to define precisely how PrP^C^ exerts its role in this signaling loop. Viewed in this manner, its contribution to NCAM1 polysialylation is not the function of PrP^C^ but may well represent the broader cellular context its function serves.

In the following paragraphs, we chose to loosely group pertinent information available in a way that reflects the principal approaches for studying a protein's function. By doing so, we will show that the proposed role is consistent with evidence gathered with any of the three aforementioned methods for studying the physiological role of a protein.

In light of the sheer volume of work published on both PrP^C^ function and NCAM1 polysialylation (for recent comprehensive reviews, see Mouillet-Richard and Vilotte, 2015 and Schnaar et al., 2014), no attempt was made to cover all facets of the pertinent literature. Instead, next to brief introductions into the biology of the key players of the PrP^C^-ST8SIA2-NCAM1 signaling loop, we will review manuscripts selected with the intent to distill common themes in the literature surrounding PrP^C^ and NCAM1 polysialylation and point the reader toward interesting facets that warrant further investigation.

### The Cellular Prion Protein

The prion protein is notorious for its causative role in rare and invariably fatal neurodegenerative diseases, known as prion diseases (Prusiner, 1995). In prion diseases, the cellular form of the prion protein (PrP^C^) undergoes profound physicochemical changes that give rise to rogue conformers known as PrP Scrapie (PrP^Sc^), a term derived from Scrapie disease in sheep, the first known prion disease. The human prion gene is coded on the short arm of Chromosome 20 (Basler et al., 1986; Lee et al., 1998). The open reading frame of the prion gene is confined to a single exon, consistent with proposed retrogene origins (Ehsani, Tao, et al., 2011) and precluding the existence of splice-isoforms. Upon removal of an N-terminal signal sequence during its translocation into the endoplasmic reticulum and replacement of a hydrophobic C-terminal signal sequence with a glycosylphosphatidylinositol-anchor (Cashman et al., 1990), the mature prion protein has a length of 208 amino acids in humans. The protein is characterized by a disordered N-terminal domain comprising a short basic motif, five imperfect octarepeats known for their ability to bind copper or other divalent cations, and a hydrophobic domain. A globular domain present at the C-terminus of PrP^C^ is stabilized by a highly conserved disulfide bridge and carries up to two N-glycans (Haraguchi et al., 1989; Riek et al., 1996; Wuthrich and Riek, 2001).

### Neural Cell Adhesion Molecules

NCAMs are members of the immunoglobulin (Ig) superfamily. The human genome codes for two NCAM paralogs, known as NCAM1 (commonly referred to as NCAM or N-CAM) and NCAM2 (also known as OCAM). The human *NCAM1* gene maps to Chromosome 11—Chromosome 9 in mice—and comprises 24 exons (Kolkova, 2010). Expression and alternative splicing of *NCAM1* gene transcripts give rise to several splice isoforms. The three most often encountered NCAM1 protein isoforms migrate under denaturing gel electrophoresis conditions with apparent molecular masses of 180, 140, and 120 kDa, hence their designation as NCAM-180, NCAM-140, and NCAM-120 (Edelman, 1984). Whereas NCAM-180 and NCAM-140 adapt a Type I transmembrane topology, NCAM-120 is, like PrP^C^, inserted into the outer leaflet of the membrane bilayer by a glycosylphosphatidylinositol-anchor. The N-terminal ectodomains of all three isoforms of NCAM1 consist of five Ig-like domains and two fibronectin Type III domains and can form homophilic (Soroka et al., 2010) and heterophilic (Nielsen et al., 2010) interactions in cis or trans.

**Box 1.** PolySia.

Sialic acids (Sia) are 9-carbon-carboxylated sugars derived from N-acetylneuraminic acid (Neu5Ac). PolySia is a linear homopolymer of Neu5Ac present on the cell surface of a subset of Gram-negative bacteria and observed attached to a small number of cell surface proteins in eukaryotic cells (Mühlenhoff et al., 1998, 2013). In mammals, polySia is made of α2,8-glycosidically linked N-acetylneuraminic acid and has been observed to vary in the degree of polymerization between 8 (the minimum number of Sia molecules required for distinguishing polySia chains from shorter oligoSia chains) and up to 90 or more sialic acid residues (Galuska et al., 2008).

**Box 2.** Mechanism of NCAM1 polysialylation.

Several studies investigated minimal requirements for NCAM1 polysialylation and revealed that short stretches of NCAM1 sequences and specific residues in its adjacent domains Ig5 and FN1 direct polySTs to NCAM1 (Fujimoto et al., 2001; Close et al., 2003). A strict requirement for tethering the Ig5-FN1 construct to the membrane was not established but membrane attachment seems to result in higher levels of polysialylation (Close et al., 2003). The crystal structure of the FN1 domain has been solved and revealed two surprising features, an acidic patch and a short α-helix not observed in other fibronectin Type III domains. Remarkably, replacement of the short α-helix with two alanine or two threonine residues misdirected polysialylation of NCAM1 from its Ig5 domain to O-linked acceptor sites within FN1 (Mendiratta et al., 2006). Taken together, these data suggested that the α-helix is not necessary for NCAM1 polysialylation but may help to orient the Ig5 domain so that its N-linked glycans can serve as polysialylation acceptor sites, possibly by promoting the formation of a kink that can be visualized in the respective region of the NCAM1 ectodomain by rotary shadow electron microscopy (Hall and Rutishauser, 1987; Becker et al., 1989). The mechanistic details of polySia attachment still remain to be revealed and may involve a secondary site of polyST interaction with the Ig5 domain (Thompson et al., 2013). However, it is increasingly apparent that a similar recognition paradigm might also underlie the polysialylation of other modular polySia substrates. In support of this notion, acidic residues in a MAM domain adjacent to the linker region carrying the O-glycan poySia acceptor sites, which are reminiscent of the aforementioned acidic patch in NCAM1's FN1 domain, might also play a role in the recognition of NRP2 as a polysialylation substrate (Bhide et al., 2016).

### PolySTs ST8SIA2 and ST8SIA4

ST8SIA2 (also known as ST8SiaII, SIAT8B, or STX) and ST8SIA4 (also known as ST8Sia IV, SIAT8D, or polyST-1 [PST-1]; Eckhardt et al., 1995; Nakayama et al., 1995; Scheidegger et al., 1995) are the only enzymes in humans and mice that can synthesize polysialic acid (polySia) with a degree of polymerization greater than 8 (Weinhold et al., 2005; Galuska et al., 2006). In the human genome, *ST8SIA2* and *ST8SIA4* genes map to Chromosome 15, Band q26 and Chromosome 5, Band q21, respectively (Angata et al., 1997). The function of these proteins is to catalyze the polycondensation of α2,8-linked N-acetylneuraminic acid building blocks to assemble linear polySia homopolymers on NCAM1 and on a very limited number of other acceptor proteins (Mühlenhoff et al., 2013). Whereas both ST8SIA2 and ST8SIA4 are strongly expressed during embryogenesis and in newborn mammals (Ong et al., 1998; Oltmann-Norden et al., 2008), ST8SIA4 represents the predominant polyST in adult brains (Hildebrandt et al., 1998; Eckhardt et al., 2000; Schiff et al., 2009). Both polySTs are Type II transmembrane proteins that carry their catalytic domains at the distal end of their ectodomain. Other domains found within polySTs are a membrane-proximal stalk domain, a transmembrane domain, and a short cytosolic domain (Nakata et al., 2006; Foley et al., 2009; Zapater and Colley, 2012).

### NCAM Polysialylation

The polySia modification (Box 1) was initially thought to be restricted to NCAM1 and the mechanism of its attachment unknown (Finne, 1982; Hoffman et al., 1982). Subsequently, polysialylation of NCAM1 was shown to occur at two N-glycan acceptor sites within its Ig-like domain 5 (Ig5; Nelson et al., 1995; Box 2). NCAM2 is not naturally polysialylated despite sharing the overall modular organization and conserved N-glycan acceptor sites with NCAM1 and being 37% identical to NCAM1 in sequence (Yoshihara et al., 1997).

Although NCAM1 represents by far the most prominent polySia protein acceptor, polySia modifications in mammals are also found on a small number of other proteins, namely, the α-subunit of the voltage-gated sodium channel (Zuber et al., 1992), CD36 (Yabe et al., 2003), neuropilin-2 (NRP2; Curreli et al., 2007), the synaptic cell adhesion molecule SynCAM1 (Galuska et al., 2010), the chemokine receptor CCR7 (Kiermaier et al., 2016), the E-selectin ligand 1 (ESL-1, gene name *GLG1*; Werneburg et al., 2016), and the two polySTs themselves (Mühlenhoff et al., 1996).

**Figure 2.**
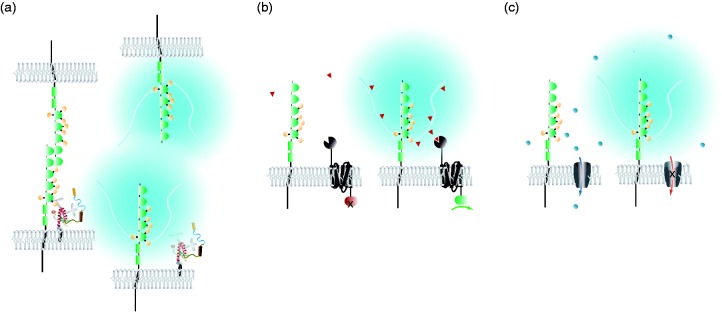
Modes of involvement of polySia-NCAM1 in cellular plasticity programs. Previously proposed categories for organizing phenotypes observed in polySia-deficient models. Note that a clean separation of these categories is often not possible as individual phenotypes may rely on more than one of the proposed mechanisms. (a) Protein interaction, (b) chemotactic guidance, (c) ion channel modulation.

How does polySia contribute to cellular function? The polySia modification of NCAM1 alters its hydrodynamic radius and confers negative charge. These properties are critical for phenotypes that have been linked to polySia-NCAM1, which can be broadly grouped into those that (a) facilitate cellular morphogenesis programs ranging from proliferation and migration to neuritogenesis and fasciculation by affecting protein interactions that mediate cell-cell or cell-substrate contacts; (b) refine cellular responses to guidance cues, or (c) modulate inputs of brain circuitry or activity of ion channels (Figure 2). Although not formally recognized as categories by which PrP^C^-related phenotypes can be grouped, examples for each of the aforementioned three types of phenotypes can also be identified in the literature surrounding PrP^C^.

Structural requirements, sequence elements, and key residues for substrate recognition and polySia transfer within polySTs are less clear. A conserved histidine residue within a C-terminal domain referred to as sialyl motif VS has been shown to be essential for activity (Kitazume-Kawaguchi et al., 2001). A polybasic region within ST8SIA4 that is expected to reside in proximity to the outer face of the lipid bilayer was proposed to contribute to NCAM1 substrate recognition. Interestingly, replacement of a particular arginine amino acid within this domain also prevented *in vitro* polysialylation of NRP2 and SynCAM1 (Zapater and Colley, 2012), an unexpected observation given that the membrane-adjacent domains of these two other known polySia carrier proteins differ fundamentally from the FN1 and 2 domains found in the respective location within NCAM1.

## PrP and polySia-NCAM1

### Protein–Protein Interactions

Several excellent reviews on protein–protein interactions that PrP^C^ (Watts and Westaway, 2007; Aguzzi et al., 2008; Rubenstein, 2012) or NCAM1 (Nielsen et al., 2010) engage in have been published before. Here, the scope will be limited to describing evidence in support of an interaction between PrP^C^ and NCAM1. Formaldehyde crosslinking of mouse neuroblastoma Neuro-2a cells, followed by affinity purification, led in 2001 to a first report on a next-neighbor relationship of PrP^C^ and NCAM1 (Schmitt-Ulms et al., 2001). Of note, the PrP^C^-NCAM1 crosslink product was not only readily detectable by denaturing gel electrophoresis but it also constituted by far the strongest crosslink signal when detection was based on PrP-directed antibodies. This observation is worth highlighting because it represents one of only a small number of data points in the literature on PrP^C^ interactions, which can be used to draw inferences on the relative abundance of proteins in proximity to PrP^C^. *In vitro* interface mapping experiments based on recombinant PrP^C^ and an NCAM1 peptide array not only corroborated the notion that the PrP^C^–NCAM1 interaction can occur through direct binding but also sketched out an interface that comprises β-strands C and C' in the two fibronectin Type III modules within NCAM1 and a nonlinear binding epitope that included the N-terminus, Helix A, and the adjacent loop domain within PrP^C^ (Schmitt-Ulms et al., 2001).

Taken together, these results were screaming for a physiological role of this interaction and suggested that NCAM1 and PrP^C^ may profoundly influence each other's biology. Enthusiasm for this line of investigation waned, however, when prion infection studies in wild-type and *Ncam1*-deficient mice revealed conclusively that NCAM1 plays no role in PrP^Sc^ replication. In subsequent years, proteomics-based discovery projects confirmed the PrP^C^–NCAM1 interaction in mouse brain (Schmitt-Ulms et al., 2004), Neuro-2a cells (Watts et al., 2009), and rat cerebellar granule cells (Farina et al., 2009) but also revealed several additional proteins, including the 37-kDa/67-kDa laminin receptor (RP21; Gauczynski et al., 2001), Na/K ATPases (Watts et al., 2009), cyclic-nucleotide phosphodiesterase (CNP; Rutishauser et al., 2009), and oligomeric forms of the amyloid precursor protein-derived Aβ-peptide (Lauren et al., 2009), in proximity to PrP^C^.

A first indication of the physiological significance of the PrP^C^–NCAM1 interaction emerged when it was reported that this interaction can occur in *cis* or *trans*, leads to the recruitment of the tyrosine kinase FYN into lipid rafts, and stimulates neuritic outgrowth (Santuccione et al., 2005). Perhaps because this work was undertaken by a preeminent NCAM research group, NCAM1 polysialylation in PrP^C^-deficient mice was also investigated at that time. Although differences in levels of polySia-NCAM1 were noted between wild-type and *Prnp*^−/−^ mice in total brain homogenates and raft fractions purified from brain or synaptic growth cones, these observations were interpreted to reflect PrP^C^ effects on the distribution of NCAM1 isoforms rather than an influence of PrP^C^ on the polysialylation of NCAM1 per se.

A new angle for investigating the significance of the PrP^C^–NCAM1 interaction came to the fore when NCAM1 emerged from an unbiased global proteomics screen as one of three proteins whose levels were not only profoundly altered in cells undergoing epithelial-to-mesenchymal transition (EMT) but were most impacted by the depletion of PrP^C^ (Mehrabian et al., 2015). This study was undertaken in NMuMG cells, a well-defined mouse model routinely used for studying this cellular transdifferentiation program. Close inspection of NCAM1 signals by Western blot analyses revealed that NCAM1 undergoes polysialylation during EMT and that PrP^C^ deficiency interferes with this posttranslational modification. The fact that polysialylated NCAM1 only exists in a small number of brain cells and is entirely absent in Neuro-2a cells, which are devoid of endogenous polySTs (Kojima et al., 1997), serves as a plausible explanation why this PrP^C^-deficiency phenotype was not observed in prior studies, which focused on these models.

NCAM1 being a direct interactor of PrP^C^, one might predict that PrP^C^ directs ST8SIA2, the polyST responsible for NCAM1 polysialylation during EMT in NMuMG cells, to its NCAM1 substrate. However, the ectopic expression of ST8SIA2 in Neuro-2a cells could restore NCAM1 polysialylation even in cell clones made PrP^C^-deficient by CRISPR-Cas9 technology. This indicated that PrP^C^ is not essential for directing ST8SIA2 to its NCAM1 substrate. Closer investigations revealed that PrP^C^ controls NCAM1 polysialylation in NMuMG cells by acting on *St8sia2* gene transcription. The precise steps involved in the PrP-dependent signaling that controls transcription of the *St8sia2* gene are not yet understood but may involve β-catenin (Mehrabian et al., 2015) as well as members of the MARCKS protein family (Mehrabian et al., 2016). Morevoer, we observed that distinct cell models exhibit a PrP-dependent upregulation or downregulation of *St8sia2* gene transcription, suggesting that input from other signaling pathways must exist (Mehrabian et al., 2015). Such an observation may, for example, be caused by subtle differences in the composition of the transcriptional complexes that assemble on the *St8sia2* promoter in response to PrP signaling. The net effect on *St8sia2* transcription may depend on whether these complexes are dominated by a repressor or activator. Data available to date also do not rule out a modulating direct effect of PrP^C^ on NCAM1 polysialylation. Finally, currently unaddressed remains the question whether the interaction between PrP^C^ and NCAM1 is essential for initiating the signaling cascade that controls the transcription of the *St8sia2* gene.

### Phenotypes of polySia-NCAM1 or PrP^C^-Deficient Models

It has been posited that an inverse correlation exists between the severity of a given gene knockout phenotype and the number of articles that will be published to describe it, a not so seriously intended statement that appears to have some merit when considering the expansive literature on PrP^C^ and polySia-NCAM1 phenotypes. Therefore, instead of attempting to provide a comprehensive review of this body of literature, we will shine a light on selected observations that illustrate possible connections between PrP^C^ and the biology of NCAM1 polysialylation.

From the wide range of examples we present, it will be apparent that expression levels of PrP and polySia-NCAM are coinciding in several cell types and anatomical structures. Having stated this, it is also important to recognize that the expression of these proteins is not strictly correlated, suggesting their expression is at least partially independently regulated in other cells.

Whereas phenotypes in PrP-deficient models were observed following PrP knockout or knockdown, there are several ways in which polySia-NCAM1 deficiency was generated in specific studies, including knockdown or knockout (a) of NCAM1 or (b) of one or both polySTs, as well as (c) digestion of polySia by endosialidases. Because NCAM1 is the predominant polySia acceptor in the brain (accounting for > 95% of this modification), phenotypes related to polysialylation in neuronal paradigms are most likely NCAM1 related. The situation is more complicated in certain hematopoietic stem cell (HSC) lineages, where polySia might be attached to one of the other polySia acceptor proteins (see earlier).

Note also that due to the passenger mutations confounder that applies to virtually all experimentally generated mouse models (Vanden Berghe et al., 2015), phenotypes observed need to be considered tentative, unless confirmed or refuted in co-isogenic models. That this concern is not merely a theoretical construct is apparent from recent reports, which drew into question previously reported roles of PrP^C^ in certain stroke paradigms (Sakurai-Yamashita et al., 2005; Spudich et al., 2005; Weise et al., 2006; Striebel et al., 2013) and the hyperphagocytosis of apoptotic cells (de Almeida et al., 2005; Nuvolone et al., 2013, 2016). In the subsequent comparison of reports on polySia-NCAM1- and PrP^C^-deficient models, we have specified the type of deficiency studied and grouped them by predominant phenotypes.

#### EMT and gastrulation

NCAM1 has been shown to be a critical regulator of EMT in a transdifferentiation paradigm of human embryonic stem cells (ESCs) acquiring mesoderm-like properties (Lehembre et al., 2008; Evseenko et al., 2010). More specifically, increases in the levels of NCAM1 and a redistribution of NCAM1 that involves its detachment from fibroblast growth factor receptors and recruitment into caveolae or raft-like domains have been recognized as early steps during EMT. The redistribution of NCAM1 coincides with stimulation of src family kinase p59Fyn, activation of focal adhesion kinase, and the assembly of integrin-mediated focal adhesions (Lehembre et al., 2008). Whereas the aforementioned study did not address the possible role of polySia in this paradigm, a related manuscript suggests that its presence may be a critical factor that determines the balance between cell-cell and cell-matrix adhesion and modulates cell migration. Thus, in a neuroblastoma tumor cell migration paradigm, the loss of polySia at sites of cell-cell contacts has been observed to translate into increased focal adhesion and attenuated cell migration (Eggers et al., 2011).

One of the best understood EMT programs in animals occurs during gastrulation and involves the detachment and migration of blastomeres during mesenchyme formation. The most dramatic PrP^C^ deficiency phenotype reported to date has been the observation of a gastrulation arrest in zebrafish embryos made to express reduced levels of PrP-1, one of two PrP^C^ orthologs in this organism, through a morpholino-based knockdown approach (Malaga-Trillo et al., 2009). Interestingly, the PrP-1-deficient embryos did not appear to suffer from an inability to initiate the EMT program. Instead, blastodermal margin cells in PrP-1-deficient embryos migrated slower during epiboly. Transplanted morphant PrP-1-deficient blastomeres also were impaired in their ability to re-establish cellular contacts and functional adherens junctions, leading to the conclusion that they cannot fully execute a cell migration program. Whereas in normal embryos, PrP-1 was shown to promote accumulation of p59Fyn at cell contacts and to increase phosphotyrosine 416 levels (a conserved epitope shared by activated src family kinases), as well as modulate the posttranscriptional E-cadherin biology, these activities were perturbed in PrP-1-deficient embryos. Importantly, the phenotype does not appear to reflect a functional specialization or idiosyncrasy of zebrafish PrP-1 because it could be rescued by the introduction of mammalian PrP^C^ (Malaga-Trillo et al., 2009).

Currently missing is information on whether the NCAM1 zebrafish ortholog plays a related role in this gastrulation paradigm. However, at least in the NMuMG mouse model of EMT, the expression of NCAM1 and PrP^C^ appears to be coordinated, with levels of both proteins substantially increasing during the first day of TGFB1-induced EMT. Moreover, in this model, PrP^C^ can be observed to enhance NCAM1 polysialylation during EMT through a signaling loop that modulates ST8SIA2 transcription (Mehrabian et al., 2015).

#### Subventricular zone, rostral migratory system, and olfactory bulb

Within the subventricular zone (SVZ), neurogenesis gives rise to glia or neuroblasts, which upon further differentiation migrate rostrally to the olfactory bulb (OB; Rousselot et al., 1995), before converting into mature neurons. PolySia-NCAM1 became a marker of adult neurogenesis research (Bonfanti, 2006; Gascon et al., 2010) ever since it emerged that its expression is not restricted to embryogenesis and early neurodevelopment but extends into adulthood within brain regions that demonstrate increased plasticity (Theodosis et al., 1991). Whereas within the adult SVZ the expression of NCAM1 can be observed in neuroblasts and glia, polySia-NCAM1 has only been observed in neuroblasts (Doetsch et al., 1997).

The first indication that NCAM1 plays a role in the rostral migratory system (RMS) was provided by the observation that the OB was reduced in size by 30% in *Ncam1*-deficient mice (Tomasiewicz et al., 1993; Cremer et al., 1994). Studies in mice deficient for both polySTs have since corroborated that polySia-NCAM1 is critical for a well-functioning RMS (Weinhold et al., 2005; Angata et al., 2007). In these mice, the cross-sectional diameter of the RMS was observed to be thicker, possibly due to cellular blockage. Consequently, the OBs were seen to be smaller, a phenotype that had also been reported following injection of the polySia-degrading enzyme endosialidase (a.k.a. endoneuraminidase; Ono et al., 1994). A more detailed characterization of the underlying structural and functional defects revealed that the smaller OBs of NCAM1 null mice are mainly due to the loss of granule cells and manifested as an impairment in the discrimination of odors, as opposed to unaltered detection threshold and short-term olfactory memory of odors (Gheusi et al., 2000).

Interestingly, mice deficient for only one of the two polySTs exhibit unaltered or only slightly reduced polySia levels in the anterior SVZ and the RMS and no gross anatomical differences in their RMS to wild-type mice (Eckhardt et al., 2000; Angata et al., 2004), suggesting a built-in redundancy is in place. Consistent with the interpretation that the reduced size OB phenotype is not caused by a failure to produce the neuronal precursors but rather reflects a defect in migration to the OB, the absence of polysialylation was accompanied by a profound increase in neuronal precursors in the RMS and SVZ (Ono et al., 1994; Hu et al., 1996; Chazal et al., 2000). Impaired RMS migration also causes accumulations of immature neurons expressing calretinin, a marker of olfactory interneurons. During postnatal development such ectopic cells appear not only in the polySia-negative RMS of mice deficient for NCAM1 or both polySTs but also in the RMS of *St8sia2* knockout mice, indicating that even minor reductions of polysialylation are sufficient to cause migration deficits of SVZ-derived neuroblasts (Röckle and Hildebrandt, 2015). Furthermore, both polyST single knockout lines have deficits of early-born interneurons in the glomerular layer of the OB and in the prefrontal cortex, which seem to be caused by defective migration during embryonic development (Kröcher et al., 2014; Röckle and Hildebrandt, 2015). Notably, these migratory deficits are observed in NCAM1- and polyST-negative mice and therefore caused by the loss of polySia-NCAM1 or polySia alone.

In contrast, the specific loss of polySia in polyST-deficient mice leads to a gain of polySia-negative NCAM1, causing severe defects of brain development that are not observed in the NCAM1 knockout, including hypoplasia or agenesis of major brain axon tracts (Weinhold et al., 2005; Hildebrandt et al., 2009). On a cellular level, experiments with neuroblastoma cells and SVZ-derived neuroblasts revealed that removal of polySia, in addition to promoting NCAM1 homophilic interactions (Johnson et al., 2005), initiates heterophilic NCAM1 interactions, which activate the MAP kinase ERK1/2 pathway and promote neuronal differentiation (Seidenfaden et al., 2003; Röckle et al., 2008).

In light of relative high levels of PrP^C^ expression observed during neurodevelopment, it has long been suspected that PrP^C^ might play a role in neurogenesis in the brain. As for polySia-NCAM, PrP^C^ expression within the SVZ was not observed in glia or mitotic cells but seems restricted to neuroblasts that reside adjacent to them (Steele et al., 2006). Indicative that the involvement of PrP^C^ in neurogenesis is not restricted to a bystander role, its presence was observed to promote the differentiation of multipotent neuroblasts into mature neurons (Steele et al., 2006).

Recently, these observations were linked by showing a role of PrP^C^ in the NCAM1-dependent differentiation of neural precursor cells in the SVZ. Specifically, SVZ-derived neuroblasts from PrP^C^-deficient mice were observed to lack the ability to respond to the presence of NCAM1 with ERK1/2 activation and neuronal differentiation, causing them instead to accumulate and cycle within the SVZ (Prodromidou et al., 2014).

The size of OBs is not altered in *Prnp*^−/−^ mice, suggesting that PrP^C^ deficiency, if it acts by influencing polySia and ST8SIA2 levels in this paradigm, does not perturb NCAM1 polysialylation altogether. Minor changes like the accumulation of calretinin-positive cells in the RMS or the reductions of specific interneuron populations as seen in *St8sia2*^−/−^ mice (Röckle and Hildebrandt, 2015) have not yet been studied in *Prnp*^−/−^ mice. However, *Prnp*^−/−^ mice were reported to exhibit deficits in odor-guided tasks that pointed to structural defects in deep granule cell layers of the OB, possibly involving their impaired communication with mitral cells via dendrodendritic synapses (Le Pichon et al., 2009).

#### Hippocampal formation

The hippocampus represents in vertebrates the primary brain region tasked with memory consolidation and spatial navigation. Detailed reports have been published that inform on the distribution of PrP^C^, polySTs, polySia, and NCAM1 within this brain region. Other documents show how the architecture of the hippocampal formation is changed in mice lacking the respective genes (Colling et al., 1997; Nacher et al., 2010). The subsequent summary of this literature will also first deal with localization before moving on to knockout phenotypes.

Both ST8SIA2 and 4 are most prominently observed in dentate granule cells (Angata et al., 1997). As analyzed in young rats, *St8sia2* mRNA is localized to cells in the subgranular zone, the innermost region of the granule cell layer and neurogenic niche of the dentate gyrus (Hildebrandt et al., 1998). Consistent with this, *St8sia2* knockouts possess a diminished number of polySia-positive cells in the subgranular zone (Angata et al., 2004). The frequency of mitotic neural progenitors was not altered (Angata et al., 2004), but many of the immature granule neurons showed aberrant location and altered morphology, suggesting a role of ST8SIA2 in their guidance and differentiation (Nacher et al., 2010). In contrast, *St8sia2* mRNA signals are broadly distributed over the entire depth of the granule cell layer (Hildebrandt et al., 1998), which is consistent with the almost complete loss of the prominent polySia immunoreactivity on mossy fibers, the unmyelinated axons projecting from the granule cells to pyramidal cells of the CA3 region of Ammon's horn (Eckhardt et al., 2000; Nacher et al., 2010). Likewise, the presence of *St8sia4* but not *St8sia2* mRNA in the pyramidal cell layer of the CA1 region (Hildebrandt et al., 1998) corresponds to the loss of polySia from the stratum lacunosum moleculare of the CA1 field in *St8sia4*^−/−^ but not *St8sia2*^−/−^ mice (Nacher et al., 2010). Resembling the pattern of hippocampal polyST expression, PrP^C^ is within this brain region most prominently expressed in dentate granule cells and in CA1 to CA3 pyramidal cells (Tremblay et al., 2007).

**Figure 3.**
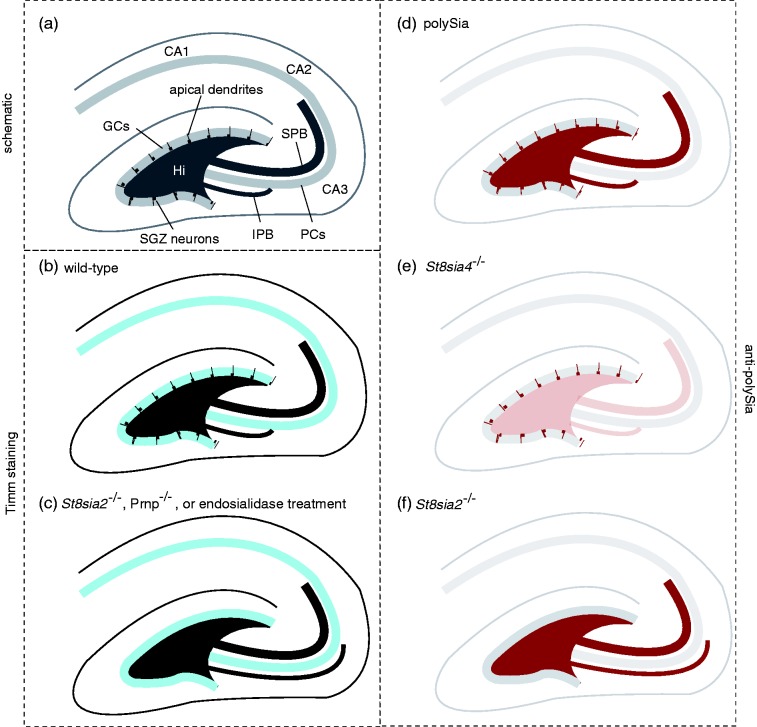
Knockout of *St8sia2* or PrP gives rise to infrapyramidal mossy fiber bundle pathfinding defect. Explanatory panel summarizing infrapyramidal mossy fiber bundle (IPB) pathfinding defect. (a) Cartoon depicting pertinent anatomical elements within hippocampus. (b) Normal IPB pathfinding observed in hippocampal formation of wild-type mice. (c) Perturbed IPB pathfinding observed following endosjalidase treatment (Seki and Rutishauser, 1998), *St8sia2* knockout (Angata et al., 2004), or *Prnp* knockout (Colling et al., 1997). (d) PolySia expression in wild-type mice. (e) Dramatic lack of polySia signals throughout the hippocampus in *St8sia4* knockout mice, except for staining of a thin layer of cells within the inner molecular layer (Nacher et al., 2010). (f) *St8sia2* knockout mice exhibit distriubtion of polySia similar to wild-type, except for reduced polySia signals in the subgranular zone (Nacher et al., 2010).

Two types of mossy fiber bundles are known, a suprapyramidal (SPB) main bundle and a smaller infrapyramidal bundle (IPB) that innervates dendrites on basal CA3 pyramidal cells and normally crosses the pyramidal cell layer to connect to the SPB within the CA3 field and in proximity to the hilus. As early as 1997, it was reported that PrP^C^-deficient mice exhibit abnormal targeting of the IPB (Colling et al., 1997). More specifically, in affected knockout mouse lines, the IPB—which can be visualized at low resolution by TIMM staining—did not cross over but instead continued to run underneath the pyramidal cell layer to the apex of its curvature (Figure 3). Significantly, since that time, only few other genes were shown to play a role in the biology that governs proper IPB targeting. Among these are *Ncam1*-knockout mice (Seki and Rutishauser, 1998) and *St8sia2*-deficient mice (Angata et al., 2004) as well as mice lacking both polySTs (Weinhold et al., 2005) and mice treated with endosialidase (Seki and Rutishauser, 1998) but not *St8sia4*-deficient mice (Eckhardt et al., 2000). Notably, the increased granulation suggestive of mossy fiber terminal sprouting as observed in the extended infrapyramidal layer of PrP^C^-deficient mice (Colling et al., 1997) is highly reminiscent to the extension of the IPB and the formation of ectopic synapses in the far CA3 region of *St8sia2*-knockout mice (Angata et al., 2004). The importance of balanced polySia synthesis during mossy fiber tract formation has been corroborated in a slice culture model, in which aberrant mossy fiber projections and formation of ectopic synapses were induced by treatment with a modified sialic acid precursor and inhibitor of ST8SIA2 activity (Vogt et al., 2012).

The mossy fiber phenotype has mostly been discussed as a deficit of lamination and fasciculation caused by the modulation of cell surface interactions. Loss of polySia could either increase fasciculation by increasing interactions between axons or reduce fasciculation by increasing interactions with the environment. Alternatively, the mossy fiber phenotype could be explained by alterations of the retraction and pruning of neuritic processes and axons, a major developmental mechanism known to contribute to neuronal maturation. It has long been known that mossy fibers first send out, then retract filamentous extensions to nearby cells during their transit through the hilar region of the dentate gyrus (Amaral, 1979). This concept was further corroborated when a careful immunohistochemical study revealed that during postnatal neurodevelopment even hippocampal formations of wild-type mice exhibit extended IPBs which, however, get pruned back around postnatal day 20 (Bagri et al., 2003).

In addition to the aforementioned players, the analysis of mice producing a loss-of-function NRP2 mutant revealed a critical role of this protein in the correct targeting of IPBs (Chen et al., 2000). Finally, a phenocopy of this IPB pathfinding defect can also be observed in mice deficient for the coreceptor of NRP2, plexin-A3 (Cheng et al., 2001), and its ligand, semaphorin-3F (Sahay et al., 2003). Thus, a model is emerging whereby pruning of IPBs not only depends on the semaphorin-3 ligand recognizing its NRP2/plexin-A3 heterodimeric receptor but may also require PrP^C^ and ST8SIA2-mediated polysialylation. Interestingly, NRP2 is a target for polysialylation but polySia-NRP2 is exclusively produced by ST8SIA4 (Rollenhagen et al., 2013) and so far has only been detected in dendritic cells (DCs), macrophages, and microglia and not in neurons (Curreli et al., 2007; Stamatos et al., 2014; Werneburg et al., 2015). PolySia, however, may modulate the local concentration of ligands that act as attractants or repellents during mossy fiber guidance or may promote the retraction of temporary axonodendritic contacts formed between mossy fibers and pyramidal cell dendrites.

Taken together, the knockout phenotypes and patterns of expression of PrP^C^ and polySTs in brain areas undergoing neurogenesis are consistent with a coordinated role of these proteins in neuroblast differentiation. And although the notion that these proteins contribute to both EMT and neuronal differentiation might be counterintuitive, it is increasingly being recognized that the morphogenetic reprogramming underlying both processes are overlapping (Itoh et al., 2013).

#### HSCs and their derivatives

The precise steps that govern the conversion of totipotent ESCs into multipotent mesoderm-committed cells are still not known. Recent gene expression profiling experiments, however, revealed the underlying shift of the transcriptome to bear yet again striking resemblances to previously established molecular signatures of cells undergoing EMT (Evseenko et al., 2010). Thus, whereas ESCs rely on E-cadherin-dependent cell-to-cell contacts and express claudins, beta-catenin, and occludin, during mesoderm-commitment, these epithelial marker proteins are replaced by the expression of mesenchymal proteins, including snail, vimentin, fibronectin, and NCAM1—note that the latter is better known as CD56 in the literature not concerned with the study of the brain. Interestingly, multipotent HSCs, which represent the main mesoderm-committed progenitors within the bone marrow of vertebrates, together with progenitors of endothelial and mesenchymal cells, smooth muscle cells, and cardiomyocytes, express ST8SIA4, but not ST8SIA2, and rely on this polyST for the expression of polySia on their cell surface (Drake et al., 2008). A recent report extended these observations by showing that ST8SIA4 is upregulated during early meso- and endoderm differentiation of human pluripotent stem cells, whereas ST8SIA2 is elevated in ectodermal cells (Berger et al., 2016).

Like NCAM1, PrP^C^ is not found on totipotent human ESCs but is expressed on HSCs expressing the marker protein CD34 (Dodelet and Cashman, 1998). In PrP^C^-deficient mice, self-renewal of HSCs is impaired leading to a gradual reduction in the proportion of multipotent long-term HSCs (Zhang et al., 2006). Because long-term HSCs serve as progenitors of myeloid and lymphoid lineage cells, another way to investigate their health is to monitor certain populations of cells derived from them by further differentiation and maturation. This approach has been taken with *St8sia4*-deficient mice which have been reported to exhibit reduced levels of the earliest thymocyte progenitors leading to an overall 30% reduction of total thymocytes. Mechanistic investigations, in which depleted T-cell progenitor reservoirs were repopulated by engraftment from wild-type or *St8sia4*-deficient mice, suggested that the absence of polySia impairs the ability of progenitors to leave the bone marrow (Drake et al., 2009).

For PrP^C^, relatively detailed characterizations of its expression within the various lineages of HSCs-derived cells are available (Isaacs et al., 2006). According to these, levels of PrP^C^ and trends of its expression are distinct for each cell type as it matures. For example, PrP^C^ expression is relatively high in monocytes and increases further as these mature into DCs but is gradually reduced upon differentiation of HSC progenitors into granulocytes. PrP^C^ is further expressed on T- and B-lymphocytes as well as natural killer cells. How polysialylation levels change as HSC progenitors commit to specific myeloid or lymphoid cell lineages is currently less well understood. However, similar to PrP, relatively high levels of polySia-NCAM1 have been observed on the cell surface of monocytes and natural killer cells (Husmann et al., 1989; Drake et al., 2008).

Interestingly, as monocytes differentiate, initially, into immature, then mature DCs, the substrate for attachment of polySia shifts from NCAM1 to NRP2 (Curreli et al., 2007). Efforts to determine the polyST responsible for NRP2 polysialylation revealed that, although DCs express both ST8SIA2 and ST8SIA4, only *St8sia4* mRNA levels were increased during DC maturation. Consistent with these observations, recent data established that polysialylation of NRP2 is indeed exclusively mediated by ST8SIA4 (Rollenhagen et al., 2013). ST8SIA4-mediated synthesis of polySia on NRP2 (Werneburg et al., 2015) and on E-selectin ligand-1 (Werneburg et al., 2016) has also been found in microglia, the resident tissue macrophages of the central nervous system (Werneburg et al., 2015). Interestingly, the polySia on non-NCAM1 protein carriers is confined to the Golgi compartment of microglia but released in response to the inflammatory activation by lipopolysaccharides, mimicking an infection by Gram-negative bacteria. Considering the anti-inflammatory activity of polySia (Shahraz et al., 2015; Werneburg et al., 2015, 2016), it can be hypothesized that this LPS-induced release is part of a negative feedback regulation of microglia or macrophage activation.

What is the functional significance of NRP2 polysialylation on the surface of DC cells? It is well known that mature DCs migrate toward secondary lymphoid organs under the guidance of specific chemokines. In particular, chemokine C-C motif ligands 19 (CCL19) and 21 (CCL21) are known to be essential in this context. Significantly, polySia on DC cells facilitates this migration in a manner that depends on the presence of a basic motif present at the C-terminus of CCL21 and, presumably, involves its interaction with the polySia negative charge cluster (Rey-Gallardo et al., 2010). This chemotaxis is strictly dependent on ST8SIA4 and not on ST8SIA2 (Rey-Gallardo et al., 2011). Originally thought to depend on polySia-NRP2, a recent study reveals that polysialylation of CCR7, the central chemokine receptor controlling immune cell trafficking to secondary lymphatic organs, is essential for the recognition of CCL21 by DC cells (Kiermaier et al., 2016). The same study also provides a mechanistic explanation why CCR7 needs polySia. CCL21 adopts an autoinhibited conformation, which is released by polySia interactions with its basic C-terminal extension.

The question arises if the ability of PrP^C^ to influence polysialylation levels extends to ST8SIA4 and non-NCAM1 substrates in certain cellular contexts. If so, it will be attractive to specifically investigate if PrP^C^ influences in immune cells the expression of ST8SIA4, thereby modulating microglia or macrophage activation or CCL21-dependend migration of DCs. Although this question has not yet been directly addressed, it was reported that transgenic overexpression of CCL21 from an insulin promoter causes chronic inflammatory disease in the pancreas that was characterized by PrP^Sc^ accumulation in prion-infected mice in this peripheral organ that in wild-type mice is spared by PrP^Sc^ infection (Heikenwalder et al., 2005). It appears as if CCL21 served in this paradigm as a chemotactic attractant which stimulated invasion of this organ by PrP^C^ expressing cells, a scenario consistent with a broader role of PrP^C^ in controlling polysialylation, transcending its more limited role in facilitating polysialylation of NCAM1.

#### Myelin repair and maintenance

Myelination of peripheral nerves, including the sciatic nerves, depends on a complex molecular dialogue between Schwann cells and neurons that is only partially understood. Schwann cells are one of several cell lineages derived from neural crest stem cells that are known to express the polysialylated form of NCAM1. In fact, NCAM1 is not merely a marker of cells in this lineage but appears to modulate several aspects of myelination, including axonal outgrowth, the formation of contacts between axons and Schwann cells, and Schwann cell differentiation (Rutishauser, 2008; Irintchev and Schachner, 2012). Early steps of the axon-Schwann cell interaction appear to be governed by a molecular biology of Schwann cells adapted from the EMT program. Consistent with this notion, not only TGFB1 but also fibronectin and tenascin, well-established marker proteins of morphogenetically active mesenchyme (Ignotz and Massague, 1986), are expressed during these early steps. NCAM1 has been observed on fasciculating axons and on non-myelinating Schwann cells until the onset of myelination when Schwann cell processes had completed up to 1.5 turns of their axon ensheathment (Martini and Schachner, 1986). A downregulation of both TGFB1 and NCAM1 expression is essential for myelination to proceed (Einheber et al., 1995). During this step, the NCAM1 pool is largely replaced by other extracellular matrix proteins, including the myelin-associated glycoprotein (Martini and Schachner, 1988), but appears to persist in the periaxonal region of larger diameter myelinated axons (Martini and Schachner, 1986). Lesioning of peripheral nerves, including nerve transection or crushing, induces TGFB1 and repopulates axon stumps with polysialylated NCAM1, facilitating proliferation of non-myelinating Schwann cell (Covault et al., 1986; Daniloff et al., 1986; Martini and Schachner, 1988). Indeed, it has been demonstrated that peripheral nerve regeneration is reduced in the absence of ST8SIA2 (Jungnickel et al., 2012; Koulaxouzidis et al., 2015) and that induced expression of polySTs in the environment promotes peripheral nerve regeneration (Gravvanis et al., 2005; Lavdas et al., 2006; Jungnickel et al., 2012). However, exquisite timing is critical for successful peripheral nerve regeneration, as a forced continuous overexpression of polySTs in Schwann cells leads to fewer regenerated myelinated axons (Jungnickel et al., 2012).

One of the most thoroughly investigated phenotypes in *Prnp*^−/−^ mice is a peripheral neuropathy that most conspicuously is characterized by a myelin maintenance defect (Bremer et al., 2010). Reminiscent of the distribution of NCAM1, PrP^C^ was observed on both the axon and ensheathing Schwann cells during peripheral nerve development, with Schwann cell expression of PrP^C^ coming to a halt once the ensheathment process is underway. Elegant genetic rescue experiments led the authors to conclude that PrP^C^ has to be expressed on the axon to prevent this demyelinating neuropathy. In the absence of PrP^C^, myelinated axons were seen to be considerably less tightly packed and the number of large diameter myelin-ensheathed axons was reduced. There also were ultrastructural differences, including loosely condensed *onion bulb* formations and a lack of bundles comprising non-myelinated axons, also known as Remak bundles, which can be seen in wild-type controls. A deficiency in the formation of Remak bundles has also been observed in the aforementioned transgenic mice, which continuously overexpress polySTs in Schwann cells (Jungnickel et al., 2012) and in laminin-deficient Schwann cells (Yu et al., 2009). Notable characteristics in the latter mice are impaired developmental expression and interactions of NCAM1 in the mutant nerves (Yu et al., 2009).

Thus, it is conceivable that the presence of PrP^C^ on peripheral axons and myelinating Schwann cells might impact myelin maintenance and peripheral nerve regeneration on capacity of its ability to interact with NCAM1 or by influencing its levels of polysialylation.

#### Circadian rhythm

Profound sleep disturbances are a hallmark of the disease in individuals afflicted with Fatal Familial Insomnia (FFI). In humans, FFI can be caused by a single amino acid change within the prion protein sequence that replaces an aspartate with an asparagine residue at Position 178 on a background of the common methionine or methionine polymorphism at PrP^C^ amino acid Position 129. Using a knock-in strategy, this disease could be modeled in mice (Jackson et al., 2013). While the exact causes for this phenotype are not known, the neural structures for time keeping are increasingly well understood. In humans and mice, a brain region that sits atop the optic chiasm, the suprachiasmatic nucleus (SCN), is known to not only regulate the sleep or wake cycle but also to act as a master conductor that harmonizes the speed of cellular clocks within the body (O'Neill et al., 2013). The insomnia phenotype observed in FFI could be caused by a mere loss of neurons within the SCN because this area, together with surrounding thalamic regions, is strongly affected in the disease. This explanation is unsatisfying, however, on account of evidence that similar sleep or wake cycle disturbances can also be observed in cases of sporadic Creutzfeldt-Jakob disease in the absence of prominent neuronal loss in this brain region (Landolt et al., 2006). A more plausible scenario, therefore, may be that PrP^C^ plays a role in the control of the sleep or wake cycle, and that mutation or its structural conversion causes a loss or perturbation of this role. PolySia-NCAM1 is expressed in adult rodent brain (Glass et al., 1994) and also subject to diurnal fluctuations during a 24-h light or dark cycle in the SCN (Glass et al., 2003), with higher and lower levels observed during the early subjective day and night, respectively (Prosser et al., 2003). Investigations of mice deficient for individual NCAM1 isoforms or all of NCAM1, and consequently its polySia modification, revealed that polySia-modified NCAM1 is critical for intact free-running circadian rhythmicity under continuous darkness (Shen et al., 1997, 2001). The knockout of all NCAM1 isoforms was shown to cause a yet more severe phenotype that included a defective activity profile during the light or dark entrainment period (Shen et al., 2001). Although the mechanism by which polySia-NCAM exerts its control of the circadian rhythm is not yet understood, a recent study proposed that an endoproteolytic polySia-carrying NCAM1 fragment might be transported to the nucleus of cells involved in circadian control to regulate the gene expression of clock-related genes (Westphal et al., 2016).

Indicative of the insomnia component observed in a subset of prion diseases reflecting a disturbance of the physiological role of PrP^C^, one of the first prominent phenotypic changes observed in *Prnp*^*−/−*^ mice was an altered circadian rhythm (Tobler et al., 1996). More specifically, two *Prnp*^*−*/*−*^ mouse lines, generated by different targeting strategies, entrained like wild-type mice to light or dark cycles but showed longer periods of activity than wild-type control mice, when subjected to continuous darkness. A follow-up investigation determined that mRNA levels of PrP^C^ fluctuate during the day in the SCN and other forebrain regions (Cagampang et al., 1999), peaking early in the phase of increased locomotor activity, together with the gene products of several other *clock-related* genes (Buhr and Takahashi, 2013).

What might be a cause for the phase shift observed in PrP^C^ and polySia-NCAM-deficient mice? Light is known to induce retinal ganglion cells to release glutamate onto SCN neurons. The increased glutamate levels, in turn, are understood to activate NMDA receptors (NMDARs). Both PrP^C^ and polySia-NCAM1 have been shown to impact NMDAR signaling, possibly, by modulating local copper concentrations (see later). In support of this model, copper has been shown to cause a delay in circadian neural activity rhythms (Yamada and Prosser, 2014). Addition of glutamate to a slice preparation of the SCN increases polySia-NCAM1 levels causing phase delays (Prosser et al., 2003). Pretreatment of this slice preparation of the SCN with endosialidase abolishes these phase delays (Prosser et al., 2003). Thus, an intricate interplay between glutamate release, the circadian control of polySia-NCAM1 and PrP^C^ levels, as well as their ability to modulate NMDA-mediated glutamate signaling might influence the circadian phase.

#### Glutamatergic signaling and long-term potentiation

Long-term potentiation (LTP) denotes a complex postsynaptic response to extracellular glutamate exposure, widely considered the cellular correlate to memory and learning. Central to LTP are the functional interplay of two types of glutamate receptors, AMPA receptors (AMPARs) and NMDARs. Briefly, when glutamate binds to AMPARs, their ion channels open causing Na^+^ influx. As the intracellular milieu becomes increasingly depolarized, inhibitory Mg^2+^ ions occluding the channels of nearby NMDARs are released, causing them to also contribute to the Na^+^ influx and allowing additional calcium ions to enter the cell. Ca^2+^-dependent cell signaling then leads to a cascade of events that, ultimately, cause more AMPARs to be embedded in the postsynaptic membrane, thereby increasing the AMPAR-NMDAR ratio and the ability of the synapse to respond to future glutamate exposure. PolySia-NCAM1 has repeatedly been shown to modulate glutamatergic signaling reliant on AMPARs and NMDARs. For example, *St8sia4*^−/−^ mice were shown to exhibit LTP impairments following stimulation of Schaffer collateral-CA1 synapses (Eckhardt et al., 2000). Indicative of available polySia levels playing a critical role in this phenotype, these CA1 synapses are known to express polySia-NCAM1 in wild-type mice and the level of impairment appeared to correlate with the gradual loss of polySia in adult mice. *Ncam1*^−/−^ mice are similarly characterized by LTP disturbances that manifest as memory deficits in hippocampus-dependent contextual fear conditioning and extrahippocampal-cued memory paradigms. Interestingly, of these two types of memory deficits, only the contextual fear deficit is shared by *St8sia4*^−/−^ mice, indicating that this adaptation is more dependent on polySia than NCAM1 itself. Consistent with this interpretation, when acute hippocampal slices from NCAM1^−/−^ mice were injected with NCAM1, the extracellular domain of polySia-NCAM1 or polySia alone, restoration of LTP was only observed with injected material containing polySia (Senkov et al., 2006). The interaction of polySia-NCAM1 appears to be specific with regard to subsets of NMDARs and AMPARs and the directionality of its modulating effect is opposite for these receptors, that is, activity promoting for AMPARs (Vaithianathan et al., 2004) and activity restricting for NMDARs (Hammond et al., 2006; Kochlamazashvili et al., 2010).

What is the physiological significance of these effects? The polySia-dependent activity restriction of GluN2B-containing NMDARs serves a useful role. It prevents the neurotoxic chronic low-level activation of these receptors and their downstream signaling pathways, possibly on the basis of it acting as a GluN2B antagonist that competes with low levels of glutamate for binding to the receptor (Hammond et al., 2006). Thus, removal of polySia was shown to translate downstream of GluN2B into higher levels of signaling through Ras and GRF1 to p38 MAPK, which, in turn, led to increased cell death. Consistent with this model, LTP and contextual fear conditioning were restored in *Ncam1*-deficient mice by inhibiting GluN2B or by scavenging free glutamate with GTP (Kochlamazashvili et al., 2010). Not surprisingly, there also is a literature that ties NMDA activation to the expression of polySia-NCAM (Butler et al., 1999; Singh and Kaur, 2009).

Ever since the first *Prnp*^−/−^ mouse models became available (Bueler et al., 1992), researchers have attempted to gauge the extent to which the loss of PrP^C^ function may contribute to synaptic deficits observed in prion diseases. Several groups reported in the mid-90s that PrP^C^-deficiency translates into impairments in LTP (Clinton et al., 1993; Collinge et al., 1994; Manson et al., 1995) and learning and memory. As with polySia-NCAM1, the presence of PrP^C^ has most often been shown to desensitize NMDARs, making them less responsive to low levels of EtOH, NMDA, kainite, or glutamate (Shyu et al., 2005; Rangel et al., 2007; Carulla et al., 2011; Llorens and Del Rio, 2012; Black et al., 2014). This protective role has, however, not remained unchallenged (Herms et al., 1995) and was, for example, not detected when the influence of PrP on LTP was investigated in hippocampal slices dissected from intercrosses of *Prnp* knockout mice and an AD mouse model, which itself exhibits an LTP defect (Calella et al., 2010). Nonetheless, the prevailing trend seems to be that protective roles of PrP^C^ can be observed in a wide range of experimental settings and extend to PrP^C^ orthologs in species as distant as zebrafish (Fleisch et al., 2013). The mechanism through which PrP^C^ ameliorates NMDAR-dependent excitotoxicity is under intense investigation but has so far not been elucidated. It has been proposed that PrP^C^ in its copper-loaded state binds to the NMDAR complex, thereby reducing the affinity of the receptor to glycine, possibly through an allosteric effect (You et al., 2012). An alternative model sees cooperative binding of PrP^C^ and copper protect NMDA receptors by promoting their S-nitrosylation (Gasperini et al., 2015). To date, neuroprotective roles of polySia-NCAM1 or PrP^C^ on NMDA receptor biology have only been considered independently. It will be interesting to explore to which degree these phenomena manifest independently or reflect a closely linked biology of PrP^C^ and NCAM1.

### Evolutionary Context

The prion gene family evolved approximately less than a half billion years ago from an ancestral gene coding for a member of the Zrt-, Irt-like protein (ZIP) family of metal ion transporters (Schmitt-Ulms et al., 2009). The evolutionary relationship between PrP^C^ and ZIP transporters acted as a catalyst in the research path that precipitated the discovery of PrP^C^'s involvement in the control of NCAM1 polysialylation. More specifically, the idea to investigate the influence of PrP^C^ on a well-known cellular plasticity program, that is, EMT, emerged from the recognition of highly similar gastrulation arrest phenotypes, which had been independently reported for ZIP6- or PrP-1-deficient zebrafish (Yamashita et al., 2004; Malaga-Trillo et al., 2009). Importantly, whereas the PrP-1-related study had noted effects of its knockdown on the maturation and subcellular distribution of E-cadherin, the ZIP6-related study attributed the gastrulation arrest phenotype squarely to a defect in EMT, thereby providing the critical impetus to explore this program in a mammalian cell model. Being mindful of evolutionary context might also hold promise for refining the model through a rigorous evaluation of evolution-related data available for the key players involved in the proposed signaling loop.

A first indication of a relationship between PrP^C^ and ZIP transporters surfaced when it was established that two of these transporters (ZIP6 and ZIP10) can be crosslinked to PrP^C^ in Neuro-2a neuroblastoma cells exposed to low levels of formaldehyde (Watts et al., 2009). During the follow-up analysis of candidate PrP^C^ interactors, striking similarities in the molecular organization of PrP^C^ and these ZIP transporters were noticed, which precipitated an investigation into whether these proteins are evolutionarily related. Subsequent bioinformatic and structural modeling analyses then led to the surprising discovery that PrP^C^ and the ectodomains of a subset of ZIP transporters indeed share considerable sequence identity and are predicted to acquire homologous folds. Spurred by these observations, additional in-depth bioinformatic analyses sealed the conclusion that PrP^C^ and ZIP transporters are members of one large protein family comprising 17 genes in humans (Schmitt-Ulms et al., 2009; Ehsani, Huo, et al., 2011). Note that the initial observation of a molecular interaction between PrP^C^ and its paralogs ZIP6 and ZIP10 might reflect a reality of ZIP transporters assembling into dimers *in vivo* (Bin et al., 2011; Pocanschi et al., 2013), that is, PrP^C^ most likely inherited and retained this property from its ancestral ZIP transporter. Whereas ZIP transporters are expressed in all branches of life, the subset of these transporters comprising a PrP-like ectodomain only evolved in the metazoan lineage (Schmitt-Ulms et al., 2009). On the basis of synteny analyses and detailed gene structure comparisons, it can be inferred that the genomic rearrangement, which led to the creation of the prion founder gene, occurred relatively early in the vertebrate lineage. Mechanistically, this event can be reconstructed to have involved a spliced ancestral ZIP transporter mRNA and most likely was based on the genomic insertion of a reverse transcribed copy of this mRNA by a retrovirus (Ehsani, Tao, et al., 2011).

There is compelling sequence evidence in the genomes of other organisms to conclude that the aforementioned steps, which precipitated the formation of the prion gene family in vertebrates, were not an isolated event but occurred more than once. For example, in the oppossum (*Monodelphis domestica*) genome, a PrP^C^-like ZIP6-related pseudogene could be identified, which is, like the *Prnp* gene, devoid of introns within its protein coding sequence and also lacks the sequence elements coding for the transmembrane domain observed in intact ZIP transporter genes (Ehsani, Tao, et al., 2011). In contrast to the prion founder gene sequence, which upon its genomic insertion became a fully functional retrogene by adapting an upstream promoter for its expression, the oppossum gene has become a non-expressed pseudogene subject to slow sequence decay (Ehsani, Tao, et al., 2011).

Similar to the subset of ZIP transporters carrying PrP^C^-like ectodomains, NCAM1 is an ancient molecule that evolved early in the metazoan lineage but there is no evidence for its polysialylation in protostomes (arthropodes and mollusks). Consistent with this assertion, the polySTs responsible for its polysialylation may have evolved around 500 million years ago (Harduin-Lepers et al., 2005; and most likely preceded the evolutionary appearance of the prion founder gene). Available evidence suggests that around that time, a gene duplication involving an ancestral sialyltransferase must have given rise to a primordial *ST8SIA2/4* polyST gene (Harduin-Lepers et al., 2008). The proposed turn of events can be deduced from the presence of an array of four *ST8SIA*-genes, including a coding sequence for an *ST8IA2/4* gene, that map to a single chromosome in lancelets (*Branchiostoma floridae*; Harduin-Lepers et al., 2008). Lancelets are members of the phylogenetic branch of amphioxi, subphylum cephalochordate, which did not participate in the two whole genome duplication events that have been credited with the emergence of separate ST8SIA2 and ST8SIA4 paralogs and were restricted to the vertebrate lineages comprising fish and tetrapods (Harduin-Lepers et al., 2008).

Around the time of chordate speciation, and most dramatically following the whole genome duplications in early vertebrates, genomes coded for many more cell surface receptors than prior to these events. The expanded repertoire of cell surface receptors and their ability to interact *in trans* with cell surface receptors on neighboring cells allowed cell-cell communication to become more refined but also must have posed an obstacle for the execution of plasticity-related tasks. Thus, for a cell to become motile, a larger number of *in trans* protein interactions had to be negotiated or broken. It has been proposed that the emergence of NCAM1 polysialylation represented an adaptation to this reality (Rutishauser, 2008). Taken together, this crude outline of evolutionary events pertinent to the formation of the PrP^C^-ST8SIA2-NCAM1 signaling loop posits that the appearance of the *Prnp* gene served as a timely and meaningful adaptation to a new challenge.

Naturally, for a cell to become motile, many other elements need to be in place. For instance, (a) stable cell-cell adhesion complexes need to be replaced with dynamic cell-substrate interactions; (b) instead of providing structural integrity, the cytoskeleton need to convey cell movement; and (c) the cell needs to have systems in place that alert it to guidance cues and inform it on when to stop moving and re-establish connections to neighboring cells. Thus, although a defect in the control of NCAM1 polysialylation may turn out to be the single most profound change observed in PrP^C^ deficiency models, this posttranslational modification is a component of a transdifferentiation program that evolved over time and requires the concerted action of many proteins. In fact, stable PrP^C^ deficiency causes the levels of several other proteins to be affected (Mehrabian et al., 2015). In particular, proteins which undergo level changes in EMT in wild-type cells are highly significantly overrepresented among the subset of proteins whose levels are altered in stable PrP^C^-deficient cells.

Consistent with the view that the involvement of the PrP^C^-NCAM1 heteromer in cellular plasticity programs did not evolve abruptly, NCAM orthologs have been linked to these programs even in cells and species whose genomes do not code for polySTs. For example, fasciclin2 (Fas2), a fruit fly (*Drosophila melanogaster*) NCAM ortholog, is required for sensory axon guidance (Kristiansen et al., 2005), motility of glial cells along axons (Silies and Klämbt, 2010), and has been shown to be essential for the coordinated developmental movement of a cluster of cells known as border cells (Szafranski and Goode, 2004). In some mammalian cancer paradigms, unpolysialylated NCAM1 might be sufficient and indispensable for a lipid-raft centered signaling program that coordinates the concomitant disassembly of E-cadherin-based adherence junctions and formation of focal adhesions as a prelude to cellular movement (Frame and Inman, 2008; Lehembre et al., 2008). In contrast to invertebrates, which do not synthesize polySia-like structures and control the cell surface presentation of NCAM orthologues by internalization and proteolytic degradation (Schuster et al., 1996; Bailey et al., 1997), the possibility to steer NCAM1 interactions by means of polysialylation provides vertebrates with an additional level of regulation, and the ratio between unpolysialylated and polysialylated NCAM1 seems to be critical for balancing cell-cell and cell-matrix adhesion as a prerequisite for the morphogenetic events during migration and differentiation (Seidenfaden et al., 2006; Eggers et al., 2011).

A similar connection to cellular plasticity programs has also been established for the subset of ZIP transporters carrying PrP^C^-like ectodomains. For instance, in addition to the aforementioned gastrulation arrest phenotype observed in ZIP6-deficient zebrafish (Yamashita et al., 2004), the fruit fly gene fear-of-intimacy, a ZIP5/6/10 ortholog (Mathews et al., 2005), was shown to be essential for the execution of a cell migration and coalescence program during gonad formation (Van Doren et al., 2003; Mathews et al., 2006) and tracheal development (Van Doren et al., 2003). Moreover, mice deficient in the ZIP13 transporter exhibit defects in bone, teeth, and connective tissue development that implicated this transporter in impaired bone morphogenetic protein or transforming growth factor signaling (Fukada et al., 2008). Similarly, ZIP14 knockout mice are characterized by stunted growth and defects in bone morphology, possibly by affecting CREB-dependent developmental signaling in chondrocytes (Hojyo et al., 2011).

**Figure 4.**
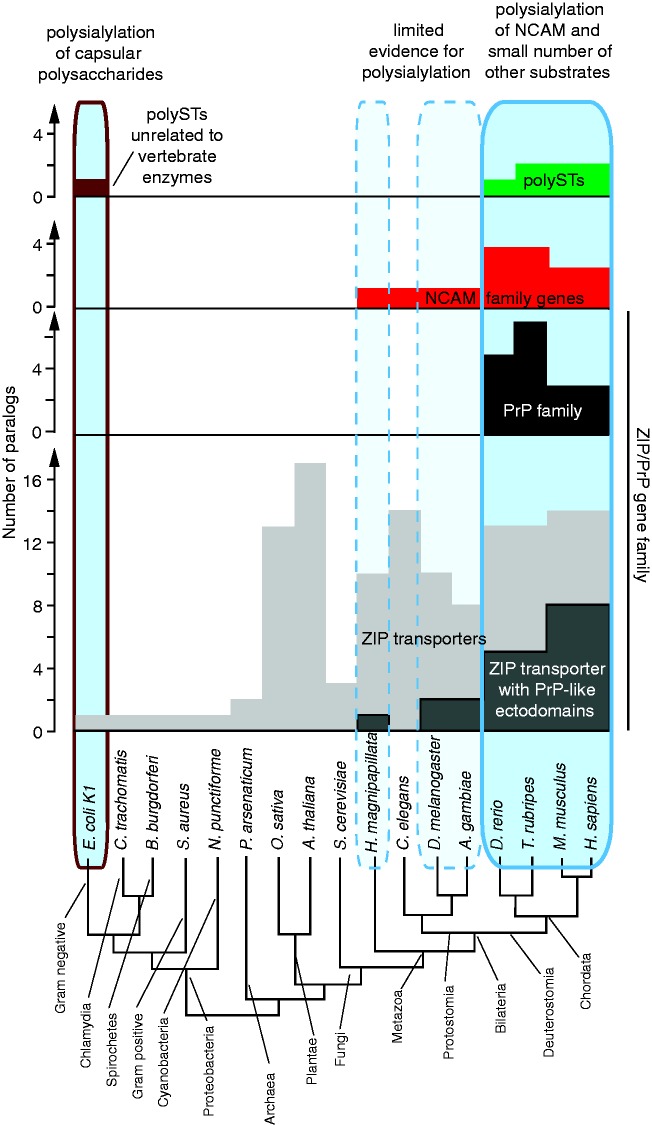
Coevolution of PrP and polySia-NCAM1. Schematic depicting numbers of paralogs within NCAM, ZIP, polyST, and PrP gene families for a wide range of species. Note that node distances and branch lengths within the simplified phylogenetic tree are not drawn to scale.

Although at least theoretically the participation of orthologs of NCAM and ZIP transporters with PrP^C^-like ectodomains in these cellular plasticity programs could be realized independent of each other, this scenario seems unlikely at this time. Existing PrP^C^ interactome data already established close proximity of PrP^C^ to NCAM1, ZIP6, and ZIP10. These data have since been corroborated by reciprocal analysis of ZIP6 interactors, which revealed NCAM1 to represent the main binding partner of ZIP6 (unpublished observation). Thus, consistent with their coinciding evolutionary emergence, NCAM1 and ZIP transporters harboring PrP^C^-like ectodomains may have evolved to collaborate during the execution of cell plasticity programs. A need for these programs materialized during metazoan development to promote a motile lifestyle. Later in evolution, with the emergence of complex body plans, elements of these programs could be adapted for developmental programs that required the migration of defined cell populations. Components required for NCAM1 polysialylation only emerged much later. These may have served the purpose to further enhance the ability of preexisting plasticity programs to cope with both an unprecedented complexity of cell surface receptor interactions and an increasing complexity of body plans. In particular, the migration of specialized cell populations in different parts of the developing body would be expected to require a more specialized toolkit to choreograph this movement in time and space, possibly requiring further differentiation of players involved. Perhaps adapting to this need, the subbranch of the ZIP transporter family comprising PrP^C^-like ectodomains expanded and diversified in the vertebrate lineage (Figure 4). It will be interesting to learn if PrP^C^-like ectodomains in ZIP transporters are able to modulate the transcription of polySTs during the execution of specific mammalian morphogenesis programs in the same way as PrP^C^.

Ironically, although this line of research was precipitated by the striking resemblance of gastrulation arrest phenotypes independently observed following PrP^C^ or ZIP6 knockdown in zebrafish embryos (Yamashita et al., 2004; Malaga-Trillo et al., 2009), it can currently not be concluded if these zebrafish defects rely on perturbed NCAM polysialylation or rely on defects in other, yet to be identified, signals that emanate from PrP^C^ or ZIP6. However, consistent with the possibility of an involvement of the PrP^C^-ST8SIA2-NCAM signaling loop in this program, the expression of ST8SIA2 (Marx et al., 2007) has been observed just prior to gastrulation during zebrafish development.

## Conclusions

This review was written with a view to provide context and perspective to a recent report, which proposed a novel role for PrP^C^ as a key player within a signaling loop that controls the polysialylation of NCAM1 during the execution of cellular plasticity programs. The role proposed for PrP^C^ reconciles within a single model insights derived from the study of (a) molecular interactions of PrP, (b) phenotypes of PrP deficiency models (Table 1), and (c) PrP^C^'s evolutionary relationship to ZIP transporters. The model emerged from a research program undertaken with no specific hypothesis regarding the biological role of PrP in mind and is the first to reconcile evidences from all main methods available for determining the function of a protein. It suggests NCAM1 and members of the branch of ZIP transporters comprising PrP^C^-like ectodomains coevolved to coordinate cellular plasticity programs early in the metazoan lineage. Approximately a half billion years later, polySTs, PrP^C^, and several additional ZIP-transporters comprising PrP^C^-like ectodomains may have evolved as an adaptation of cellular plasticity programs to the emergence of increasingly complex cell surface proteomes and body plans. The model predicts the existence of a coordinated biology of PrP^C^ and ST8SIA2 in the polysialylation of NCAM1. Because there is no shortage of tools and prior independent knowledge available for its key players, the model should prove to be exquisitely testable and offers a multitude of angles for further investigation. It is hoped that these investigations will generate answers to critical questions that relate to the role of not just PrP^C^ but also NCAM1 and the respective ZIP transporters in the context of cancer and neurodegenerative disease.

**Table 1.** Similarities Among PrP- and PolySia-NCAM-Related Phenotypes.

Phenotype	PrP-related observations	PolySia-NCAM1 related observations
1. Circadian rhythm	a. ko mice exhibit altered circadian rhythm under continuous darkness (Tobler et al., 1996) b. Diurnal fluctuations of PrP mRNA (Cagampang et al., 1999)	Critical for intact free-running circadian rhythmicity under continuous darkness (Shen et al., 1997; Shen et al., 2001) Diurnal fluctuations of polySia-NCAM1 levels (Glass et al., 2003)
2. Mossy fiber pathfinding	ko mice exhibit pathfinding defect of infrapyrimidal bundle, causing it to run underneath pyramidal cell layer (Colling et al., 1997)	NCAM1 ko mice and *St8sia2* ko mice exhibit pathfinding defect of infrapyrimidal bundle, causing it to run underneath pyramidal cell layer (Seki and Rutishauser, 1998; Eckhardt et al., 2000; Angata et al., 2004)
3. Neurogenesis	Observed within adult subventricular zone in neuroblasts; its presence promotes their differentiation into mature neurons (Steele et al., 2006)	Marker of adult neurogenesis research; observed within adult subventricular zone in neuroblasts (Doetsch et al., 1997; Bonfanti, 2006; Gascon et al., 2010)
4. LTP and voltage-gated ion channels	a. ko mice exhibit deficits in LTP (Collinge et al., 1994; Curtis et al., 2003; Rangel et al., 2009) b. Promotes AMPARs (Watt et al., 2012) c. Restricts activity of NMDARs (Khosravani et al., 2008)	Promotes LTP (Senkov et al., 2006) Promotes AMPARs (Senkov et al., 2012) Restricts activity of NMDARs (Kochlamazashvili et al., 2010)
5. Hematopoietic stem cells (HSCs)	a. Not found on totipotent embryonic stem cells (ESCs) but expressed on HSCs (Dodelet and Cashman, 1998) b. ko mice exhibit impaired HSC self-renewal (Zhang et al., 2006) c. Expression is high in monocytes dendritic cells (DCs), T- and B-lymphocytes and natural killer cells (NK) (Isaacs et al., 2006) d. Neuropilin is a candidate PrP interactor (Watts et al., 2009)	Not found on totipotent embryonic stem cells but expressed on HSCs (Drake et al., 2008) *St8sia4*-ko mice exhibit 30% lower levels of thymocytes (Drake et al., 2009) Expression is high in monocytes and NK cells; As monocytes differentiate into DCs, the substrate for polySia attachment shifts from NCAM1 to neuropilin (Curreli et al., 2007; Rey-Gallardo et al., 2011)
6. Myelin repair	a. Expressed on axon and nonmyelinating Schwann cells (Bremer et al., 2010) b. ko causes peripheral neuropathy characterized by a myelin maintenance defect (Bremer et al., 2010)	Expressed on axons and non-myelinating Schwann cells (Martini and Schachner, 1986; Nait Oumesmar et al., 1995); Implicated in regeneration of lesioned or chemically demyelinated peripheral nerves (Covault et al., 1986; Daniloff et al., 1986; Martini and Schachner, 1988; Nait Oumesmar et al., 1995)
7. Dentin abnormalities	ko mice exhibit dentin structure defects (Schneider et al., 2007)	NCAM1 levels in progenic dental pulp correlate with number of dentin producing active odontoblasts (Ustiashvili et al., 2014)

*Note*. Modified from Mehrabian et al. (2015).
